# JAK inhibition with tofacitinib rapidly increases contractile force in human skeletal muscle

**DOI:** 10.26508/lsa.202402885

**Published:** 2024-08-09

**Authors:** Joseph B Shrager, Ryan Randle, Myung Lee, Syed Saadan Ahmed, Winston Trope, Natalie Lui, George Poultsides, Doug Liou, Brendan Visser, Jeffrey A Norton, Shannon M Nesbit, Hao He, Ntemena Kapula, Bailey Wallen, Emmanuel Fatodu, Cheyenne A Sadeghi, Harrison B Konsker, Irmina Elliott, Brandon Guenthart, Leah Backhus, Roger Cooke, Mark Berry, Huibin Tang

**Affiliations:** 1 Division of Thoracic Surgery, Department of Cardiothoracic Surgery, Stanford University School of Medicine, Stanford, CA, USA; 2 VA Palo Alto Healthcare System, Palo Alto, CA, USA; 3 Department of Surgery, Stanford University School of Medicine, Stanford, CA, USA; 4 Department of Biochemistry, University of California, San Francisco, CA, USA

## Abstract

Reduction in muscle contractile force associated with many clinical conditions incurs serious morbidity and increased mortality. Here, we report the first evidence that JAK inhibition impacts contractile force in normal human muscle. Muscle biopsies were taken from patients who were randomized to receive tofacitinib (n = 16) or placebo (n = 17) for 48 h. Single-fiber contractile force and molecular studies were carried out. The contractile force of individual diaphragm myofibers pooled from the tofacitinib group (n = 248 fibers) was significantly higher than those from the placebo group (n = 238 fibers), with a 15.7% greater mean maximum specific force (*P* = 0.0016). Tofacitinib treatment similarly increased fiber force in the serratus anterior muscle. The increased force was associated with reduced muscle protein oxidation and FoxO-ubiquitination–proteasome signaling, and increased levels of smooth muscle MYLK. Inhibition of MYLK attenuated the tofacitinib-dependent increase in fiber force. These data demonstrate that tofacitinib increases the contractile force of skeletal muscle and offers several underlying mechanisms. Inhibition of the JAK-STAT pathway is thus a potential new therapy for the muscle dysfunction that occurs in many clinical conditions.

## Abstract (precis)

Inhibition of JAK activity with tofacitinib rapidly increases human skeletal muscle fiber force by up-regulating smooth muscle myosin light chain kinase, offering a new treatment for muscle weakness.

## Introduction

Normal skeletal muscle contractility is essential for mobility, respiration, mastication, and other activities that sustain life. The diaphragm is perhaps the only skeletal muscle frankly critical to life as it is the primary respiratory muscle, generating ∼75% of the negative pressure required for inspiration. Many medical conditions including cancer (Fearon et al, 2011), renal failure (Rao et al, 2018), metabolic syndrome (Nishikawa et al, 2021), disuse (including ventilator-induced diaphragm dysfunction) (Levine et al, 2008; Nunes et al, 2022), chronic respiratory diseases (Jaitovich & Barreiro, 2018), primary neuromuscular diseases, and even normal aging (Cruz-Jentoft & Sayer, 2019) can result in weakness of skeletal muscles, effecting morbidity and premature death. Whereas a drug that prevents or ameliorates muscle weakness, without incurring its own substantial toxicities, would likely have a substantial impact on patient outcomes, no such drug is currently available.

The JAK-STAT signaling pathway has been shown to impact skeletal muscle. Chronic activation of JAK-STAT promotes skeletal muscle wasting and/or weakness (Belizário et al, 2016), whereas inhibition of JAK-STAT has beneficial effects on skeletal muscle in rodent models of ventilator-induced diaphragm dysfunction, critical illness myopathy, cancer cachexia, and denervation (Bonetto et al, 2012; Smith et al, 2014; Silva et al, 2015; Tang et al, 2015; Huang et al, 2020; Addinsall et al, 2021). The effect of JAK-STAT inhibitors on normal *human* skeletal muscle function has not, to our knowledge, been reported. There have been a few studies where JAK inhibition was successfully used to treat the muscle weakness associated with dermatomyositis, by reducing the inflammatory process underlying that disease (Ladislau et al, 2018; Kim, 2021; Kim et al, 2021; Yu et al, 2021; Huang et al, 2023; Zhang et al, 2023). Note, however, that this is a concept that is essentially unrelated to a potential use of JAK inhibition to improve the strength of *normal* skeletal muscle, which is studied herein.

Several compounds that inhibit JAK-STAT are FDA-approved and in clinical use for the treatment of various inflammatory disorders. Among these, tofacitinib (Xeljanz, Pfizer, Inc.) is a small-molecule (312.4 D) oral agent approved for the treatment of rheumatoid arthritis, psoriatic arthritis, and ulcerative colitis (Dhillon, 2017; Varyani et al, 2019; Sarabia et al, 2022) and also used in some cases of dermatomyositis (Paik et al, 2021). Anti-inflammatory treatment with tofacitinib was also recently demonstrated to reduce mortality and respiratory failure in COVID-19 patients (Guimarães et al, 2021; Hayek et al, 2021; Maslennikov et al, 2021). Tofacitinib binds to JAK proteins with a potency order JAK1 > JAK3 > JAK2 > Tyk2 and inhibits JAK-dependent phosphorylation/activation of STAT proteins, which subsequently prevents the translocation of STAT proteins into the nucleus and thereby suppresses JAK-STAT–dependent regulation of gene transcription.

To examine the impact of JAK inhibition on normal human skeletal muscle, we used the available muscle biopsies taken from patients who were participating in a prospective, randomized, double-blind clinical trial. Patients were administered tofacitinib or placebo for 48 h before the surgical procedure esophagectomy, and then diaphragm and serratus anterior muscle biopsies were taken at the start of the operation, shortly after the induction of anesthesia (Shrager et al, 2021). We report here that the maximum contractile force of individual muscle fibers of both the diaphragm and serratus anterior muscles was significantly higher in the tofacitinib versus the placebo groups. JAK-STAT inhibition therefore appears to have great promise as an effective treatment for clinical weakness of the diaphragm and other skeletal muscles.

## Results

### Patient enrollment and analysis

**Figure 1.**
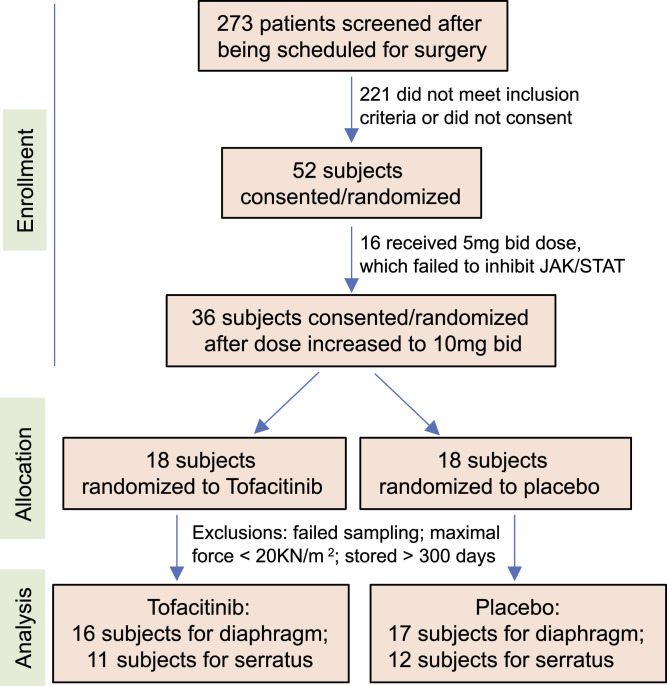
CONSORT diagram shows the flow of subject enrollment, allocation, and analysis.

The CONSORT diagram (Fig 1) shows the subjects’ flow through enrollment, allocation, and analysis. Briefly, after screening subjects based on the inclusion/exclusion criteria (detailed in the Materials and Methods section), the consented subjects were randomly allocated to receive a placebo or 10 mg of tofacitinib, twice daily (five total doses) during the 48 h immediately before a surgical procedure. Then, during the procedure, diaphragm and serratus anterior muscle biopsies were taken. Single muscle fiber force was then measured to examine the difference in fiber force between the subjects who received placebo versus subjects who received tofacitinib.

### Effectiveness of randomization

Table 1 demonstrates that the 33 subjects randomized to tofacitinib and placebo were evenly matched on all variables that one might anticipate could influence muscle biology/function. Table S1 summarizes the clinical information for all subjects.

**Table 1.** Patient characteristics.

Characteristic	Overall (n = 33^a^)	Tofacitinib (n = 16^a^)	Placebo (n = 17^a^)	*P*-value^b^
Age, years	68 (57, 70)	69 (56, 76)	68 (57, 70)	0.8
Body mass, kg*	76 (63, 90)	81 (67, 93)	73 (56, 83)	0.2
				
Sex				0.4
Female	6 (18%)	4 (25%)	2 (12%)	
Male	27 (82%)	12 (75%)	15 (88%)	
Diabetes	5 (15%)	3 (19%)	2 (12%)	0.7
Smoking status				0.5
Current	2 (6.1%)	0 (0%)	2 (12%)	
Non-current smoker	31 (94%)	16 (100%)	15 (88%)	
COPD/emphysema	3 (9.1%)	0 (0%)	3 (18%)	0.2
Esophageal cancer	28 (85%)	14 (88%)	14 (82%)	>0.9
pStage				0.9
Stage 0	2 (6.1%)	0 (0%)	2 (12%)	
Stage 1	10 (30%)	5 (31%)	5 (29%)	
Stage 2	2 (6.1%)	1 (6.3%)	1 (5.9%)	
Stage 3	13 (39%)	7 (44%)	6 (35%)	
Unspecified	6 (18%)	3 (19%)	3 (18%)	
Induction chemotherapy	23 (77%)	12 (80%)	11 (73%)	>0.9
Induction radiation	22 (73%)	11 (73%)	11 (73%)	>0.9

a n (%), median (IQR).

b Wilcoxon rank sum test; Fisher’s exact test.

* Weight loss of greater than 5% was an exclusion criterion for the study.

Abbreviations: COPD, chronic obstructive pulmonary disease; pStage, pathologic stage.

Table S1. Clinical information for all subjects.

### 48 h of oral tofacitinib suppresses the activity of the JAK-STAT signaling pathway in human diaphragm muscle

**Figure 2.**
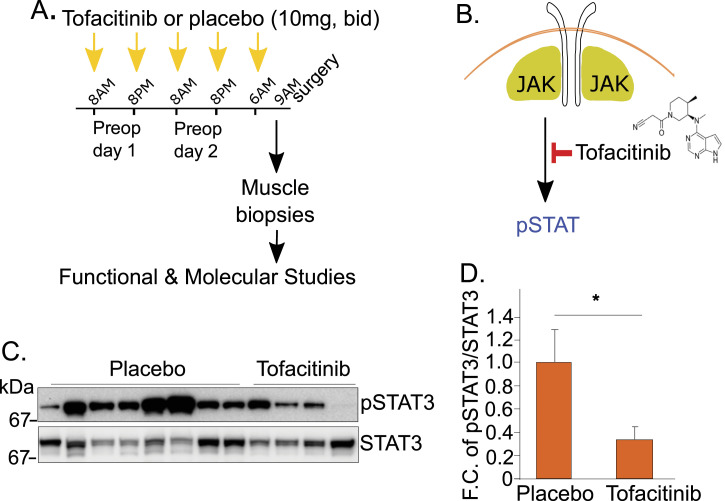
48 hs of oral tofacitinib inhibits JAK-STAT activity in the human diaphragm muscle. **(A)** Diaphragm biopsies were taken at the start of esophagectomy after five doses of tofacitinib or placebo taken orally over the 48 h before surgery. **(B)** Tofacitinib inhibits JAK-STAT signaling through inhibition of JAK-dependent phosphorylation of STAT proteins. **(C)** Suppression of JAK-STAT signaling is revealed by Western blot showing the phosphorylation levels of STAT3 (12 consecutive subjects). **(D)** Quantitation of the relative levels of phosphorylated STAT3 (pSTAT3) over total STAT3 protein (STAT3) in all specimens tested. Placebo: n = 12; tofacitinib: n = 9.

**Figure S1.**
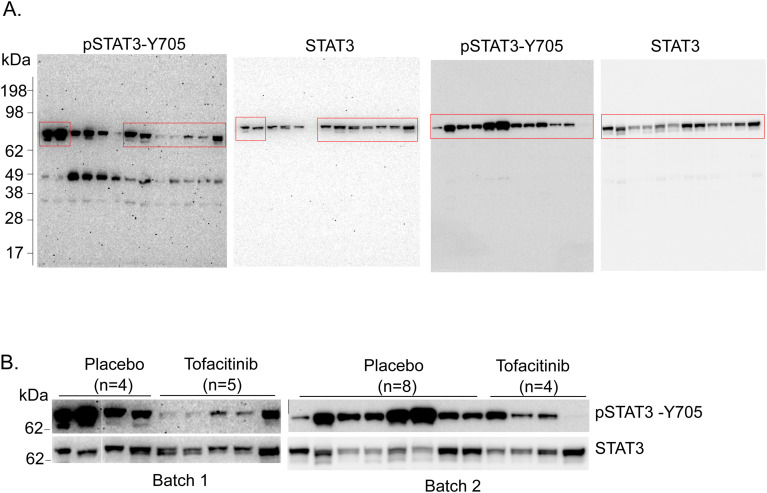
Original Western blotting results for the data shown in Fig 2C and D. **(A)** Original Western blotting results. Note that we measured the amounts of STAT3 and pSTAT3 in 2 batches of biopsies (9 in batch one and 12 in batch 2). Bands in the red boxes are samples from the actual trial subjects. **(B)** Samples which were run on the same gel using specimens from patients sampled before the trial, and thus not randomized between tofacitinib and placebo, are not highlighted in red, and these have been removed in (B), below. **(B)** The final Western blot results from each batch, with the non-randomized, non-red-highlighted specimens removed, are shown in the lower panels. In the main manuscript Fig 2C, we have shown only Batch 2, for clarity. However, both Batch 1 and Batch 2 results were quantitated to arrive at the fold change of pSTAT3/STAT3 in tofacitinib-treated versus placebo groups shown in Fig 2D.

After administration of five doses of tofacitinib or placebo, biopsies from the diaphragm and serratus muscles were taken ∼1.5 h after initiation of mechanical ventilation (MV) in tofacitinib and placebo subjects (Fig 2A and B). We examined levels of phosphorylated STAT3 using Western blotting to probe the activity of JAK-STAT signaling in the muscle biopsies. STAT3’s phosphorylation (i.e., pSTAT3), normalized to total STAT3 protein, was significantly suppressed in the tofacitinib group versus placebo (Figs 2C and D and S1A and B). Thus, short-term (48 h) administration of oral tofacitinib 10 mg twice daily effectively inhibits the activity of the JAK-STAT signaling pathway in the diaphragm.

### 48 h of oral tofacitinib increases the contractile force of both diaphragm and serratus anterior myofibers

**Figure 3.**
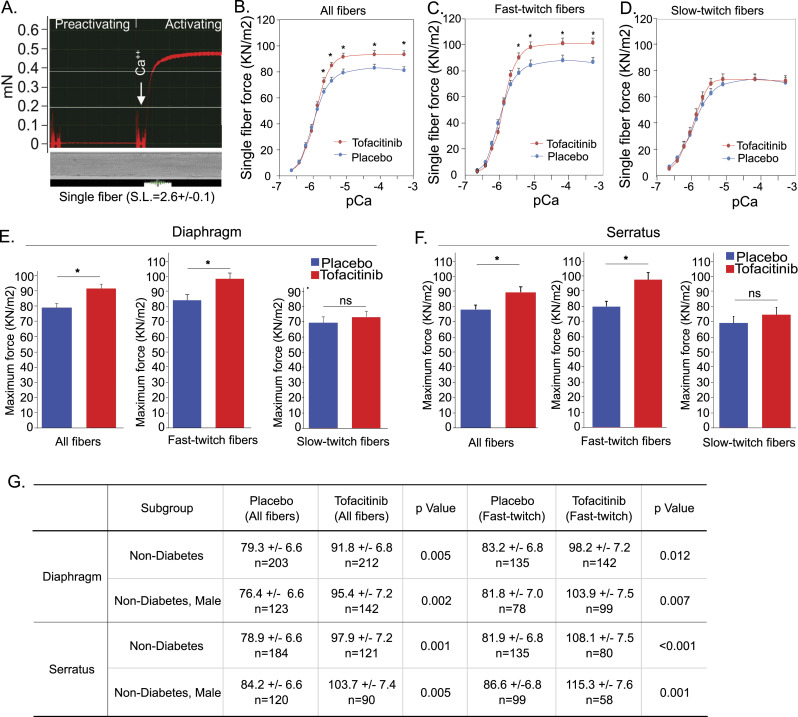
48 h of oral Tofacitinib increases the contractile force of human diaphragm and serratus anterior fibers. **(A)** A representative ex-vivo, single human diaphragm fiber contractile force curve induced by exogenous calcium. Sarcomere length is set at 2.6 ± 0.1 μm. **(B)** Force-calcium curves demonstrate that the specific force of single-diaphragm fibers in all-fiber types combined is higher in the tofacitinib group than in the placebo group at multiple calcium concentrations (from pCa 5.4 to pCa 3.3). Tofacitinib: n = 248; placebo: n = 238; *P < 0.05. **(C)** Force-calcium curves demonstrate that the specific force of single diaphragm fibers in fast-twitch fibers is higher in the tofacitinib group than the placebo group at multiple calcium concentrations (from pCa 5.4 to pCa 3.3). Tofacitinib: n = 170; placebo: n = 160; *P < 0.05. **(D)** Force-calcium curves demonstrate that the specific force of single diaphragm fibers in slow-twitch fibers is not altered significantly in the tofacitinib group compared with the placebo group at each of the calcium concentrations (from pCa 5.4 to pCa 3.3). Tofacitinib: n = 59; placebo: n = 64; *P < 0.05. **(E)** Mean maximum specific force of single diaphragm fibers in both all-fiber and fast-twitch types is significantly higher from tofacitinib-treated subjects than placebo-treated subjects (measured at pCa 5.1). Tofacitinib: n = 248 all fiber, n = 170 fast fibers; placebo: n = 238 all fiber, n = 160 fast fibers; *P < 0.05. Mean maximum specific force of single diaphragm fibers in slow-twitch type is not significantly altered in tofacitinib-treated subjects compared with placebo-treated subjects (measured at pCa 5.1). Tofacitinib: n = 59; placebo: n = 64; *P = 0.3. **(F)** Mean maximum specific force of single serratus anterior myofibers in both all-fiber and fast-twitch types is significantly higher from tofacitinib-treated subjects than placebo-treated subjects (measured at pCa 5.1). Tofacitinib: n = 191 all fiber, n = 123 fast fibers; placebo: n = 234 all fiber, n = 174 fast fibers; *P < 0.05. Mean maximum specific force of single serratus anterior myofibers in slow-twitch types is not significantly altered in tofacitinib-treated subjects than in placebo-treated subjects (measured at pCa 5.1). Tofacitinib: n = 58 fibers; placebo: n = 47 fibers; *P = 0.2. **(G)** Fiber force analyses in subgroups of the randomized subjects. In both non-diabetic subjects and in non-diabetic male subjects, fiber force (both in all-fiber and fast-twitch type fibers) is significantly higher in the tofacitinib-treated groups, in both diaphragm and serratus muscles.

**Figure S2.**
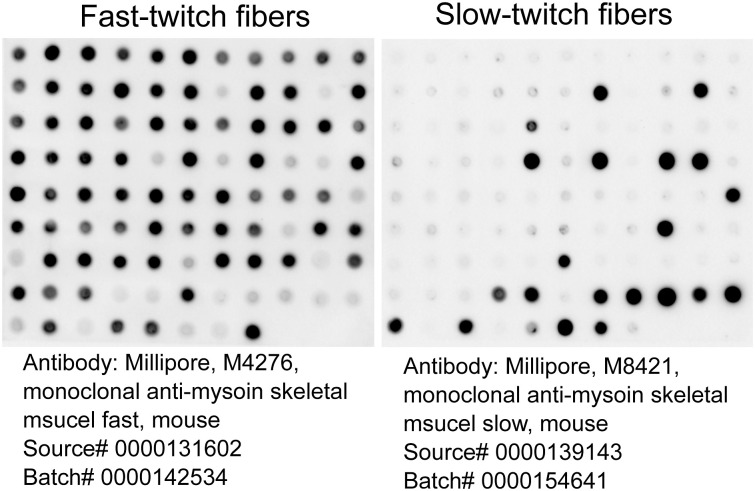
Representative images of fiber typing with dot blotting analysis. Single-fiber lysates from a total of 96 fibers were extracted after recovering from fiber force measurement. Lysates were then duplicated onto two separate nitrocellulose membranes. The fiber types were detected with fast-twitch specific antibody (left) and slow-twitch specific antibody (right), respectively. Antibody information is indicated below the images.

The contractile force of individual myofibers from the diaphragm and serratus anterior muscle was measured. As shown in Fig 3A, exogenous calcium activates the skinned individual fibers, inducing contraction. Fiber types were determined after force measurement with specific antibodies against fast- and slow-twitch fibers. Representative fiber typing results are shown in Fig S2. Based on this fiber typing, results of single-fiber force were able to be grouped into three categories of fibers: all fibers, slow-twitch fibers, and fast-twitch fibers.

To identify the calcium concentration that would induce maximal fiber contractile force, calcium response curves (i.e., force-pCa curve) were constructed using diaphragm myofibers. Fig 3B–D show force increasing with calcium concentration along a curve. After an initial rapid induction of fiber force as the calcium concentration increases, fiber force plateaus at calcium concentration from pCa 5.1 to pCa 3.3. A significant difference in the fiber contractile forces between the tofacitinib- and placebo-treated subjects (higher in the tofacitinib group) is seen at the indicated calcium concentrations in all fibers and fast-twitch fibers (Fig 3B and C), but no difference is seen in slow-twitch fibers (Fig 3D).

For all fiber types pooled, the mean maximum specific force (consistently reached at pCa 5.1 exogenous calcium) was 15.7% and 14.7% higher in the tofacitinib-treated versus placebo groups, in the diaphragm and serratus anterior fibers, respectively (*P* < 0.01) (Fig 3E and F). A similar result was seen in the group of only fast-twitch muscle fibers (Fig 3E and F), at 16.5% and 22.0% greater in the tofacitinib-treated diaphragm and serratus muscle fibers, respectively (*P* < 0.01). However, there was no significant difference between the experimental and placebo groups in the maximal contractile force of slow muscle fibers, either in the diaphragm or the serratus anterior muscle (Fig 3E and F).

### Subgroup analyses of muscle contractile force in the tofacitinib versus placebo groups

The random allocation to receiving tofacitinib or placebo that was used in this study is the ideal method to minimize baseline differences between experimental and placebo groups, and indeed, Table 1 demonstrates that the randomization was effective, with no significant differences between the groups. However, to gain even further assurance regarding the possible role of confounding baseline factors in the subjects, we also compared the maximal forces created by fibers from the tofacitinib versus placebo groups within various more homogeneous subgroups of subjects.

The largest identifiable subgroup with homogeneity on a baseline characteristic that could be hypothesized to impact muscle force response to JAK inhibition is non-diabetic subjects, with n = 28 (n = 15 placebo and n = 13 tofacitinib), and n = 203 and n = 212 fibers in the diaphragm, respectively. In this non-diabetic subgroup, the mean maximal fiber force (both all fibers and the fast-twitch fibers only) was significantly higher in the tofacitinib group than the placebo group, in both the diaphragm and serratus muscles (Fig 3G). In trying to identify a sizeable, even more homogeneous, relevant subgroup, we looked at male non-diabetic subjects. In this group as well, the fiber force is significantly higher in the tofacitinib group than the placebo group, both for all fibers and fast-twitch fibers only, in both the diaphragm and serratus anterior muscle (Fig 3G). Other subgroups that would be potentially of interest had insufficient fiber numbers to provide meaningful information, and for the same reason, we are unable to carry out additional multivariate analyses to determine the combined effects of several factors. Thus, in both the primary randomized trial cohorts and variable-defined subgroups with more homogenous backgrounds, tofacitinib increased muscle fiber force generation.

### Bootstrapping results

**Figure 4.**
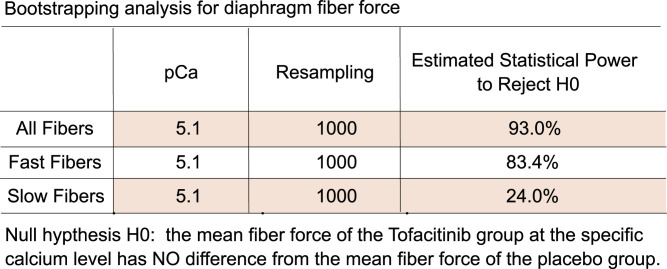
Bootstrapping analysis using random sampling with replacement. The computational resampling was repeated for 1,000 iterations from the fiber force dataset of the diaphragm. These results indicate that the null hypotheses of no difference between the placebo and tofacitinib groups can be rejected with high statistical power with the n used in this study.

Post hoc bootstrapping was performed to estimate the power of our data to reject the null hypotheses with regard to the measured differences in maximal muscle fiber force (Fig 4). As shown, in both the all-fiber and fast-twitch fiber groups, but not in slow-twitch fibers, the analyses show that the power to reject the null hypothesis (H0) was above 80%. There was a 93% estimated power in the all-fiber group, and an 83% estimated power in the fast-fiber group, to reject the null hypothesis that there is no difference between the maximal forces created by the tofacitinib- and placebo-treated fibers. There is only a 24% estimated power to reject the null hypothesis in the slow-fiber group, indicating that the finding of no difference in the tofacitinib- versus placebo-treated slow fibers is likely true.

### Tofacitinib alters the molecular signature and transcriptionally down-regulates genes participating in protein ubiquitination/proteasome in skeletal muscle

**Figure 5.**
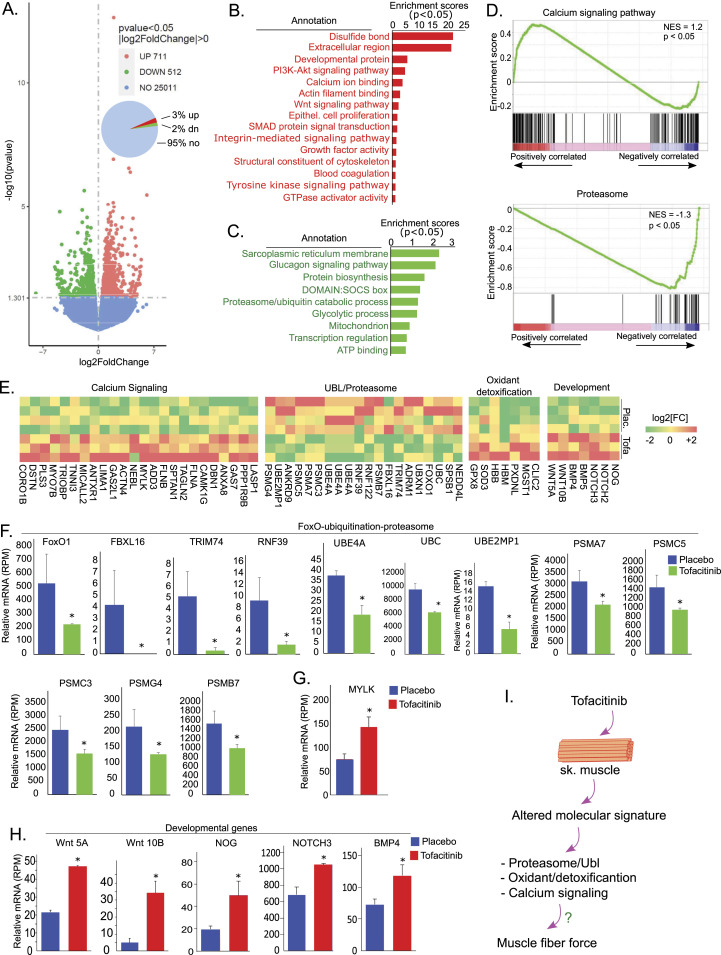
Tofacitinib transcriptionally up-regulates genes that control muscle contraction and down-regulates genes participating in protein ubiquitination in human diaphragm. **(A)** Tofacitinib treatment altered the transcriptomic profiling in human diaphragm, up-regulating 3% (red) and down-regulating 2% (green) of genes detected by RNA sequencing, shown here on volcano plot. **(B, C)** Tofacitinib up-regulated (red) and down-regulated (green) molecular events in skeletal muscle. Note that induced events included oxidoreduction, and calcium and actin filament binding, and suppressed events included proteasome/ubiquitin processes ((C), green). Molecular events were analyzed by DAVID bioinformatics and ranked by enrichment scores. **(D)** Representative KEGG signaling pathways among the differentially expressed genes. Consistently, GSEA analysis revealed enrichment of up-regulated genes in calcium signaling pathways and down-regulated genes in the proteasome pathway. **(E)** Heatmap of the differentially expressed genes in calcium, muscle contraction, and proteasome signaling pathways. **(F)** Quantitation of degree of down-regulation of genes that underlie protein ubiquitination and proteasome. *P < 0.05. **(G, H)** Quantitation of degree of up-regulation of MYLK mRNA (G) and development-regulatory genes (H) in diaphragm muscle. *P < 0.05. All transcriptomic experiments tofacitinib: n = 3 random subjects; placebo: n = 4 random subjects. **(I)** A summary of tofacitinib-altered genes and their hypothetical roles in muscle force generation.

To examine the mechanisms underlying the tofacitinib-induced increase in muscle contractile force, we first performed an unbiased investigation of transcriptome changes in response to tofacitinib treatment with RNA sequencing on diaphragm extracts from the tofacitinib (n = 3 subjects) and placebo (n = 4 subjects) groups. This global transcriptomic analysis revealed 711 genes significantly up-regulated and 512 genes down-regulated in diaphragm muscle with tofacitinib treatment versus placebo (Fig 5A). DAVID bioinformatics analysis revealed that the up-regulated genes are enriched in several molecular events that may be relevant to muscle contractility, including oxidation–reduction (e.g., disulfide bond), extracellular region (e.g., integrin-mediated signaling), and contraction (e.g., calcium ion binding, actin filament binding) (Fig 5B); the down-regulated genes are mainly clustered in sarcoplasmic reticulum membrane, proteasome/ubiquitin protein modification/catabolic process, mitochondria, and glucose and protein metabolism categories (Fig 5C). To further validate the altered molecular events, we also performed global signaling mining with gene set enrichment analysis. The complete list of the significantly altered KEGG-signaling pathways is shown in Table S2. Interestingly, signaling pathways relevant to calcium and contraction were activated, whereas proteasome was suppressed (Fig 5D), consistent with the findings of the DAVID bioinformatics analysis.

Table S2. Complete list of KEGG-signaling pathways altered in diaphragm extracts by Tofacitinib therapy.

The differentially expressed genes clustered in these pathways are shown in the heatmap (Fig 5E). Quantitative changes in representative genes in these pathways are shown in Fig 5F and G. The expression of genes participating in the ubiquitination and proteasome systems (responsible for muscle protein functional modification and degradation) are down-regulated by tofacitinib. These include *FoxO1* and genes underlying ubiquitin-proteasome activity (F-box proteins [e.g., FBXL 16], TRIM proteins [e.g., TRIM74], RING finger proteins [e.g., RNF39], ubiquitin conjugation factors [UBEs and UBC], and proteasome subunits [PSMs]) (Fig 5F). On the other hand, the smooth muscle type of myosin light chain kinase (*MYLK*), a regulatory gene involved in calcium signaling and smooth muscle contraction, is among the genes that are significantly up-regulated by tofacitinib administration (Fig 5G). We also observed that genes that regulate development, such as *Wnt*, *Noggin* (*NOG*), and bone morphogenetic proteins, are activated in skeletal muscle by tofacitinib, revealing that tofacitinib treatment also activates genes that normally express during development, although the direct involvement of these genes in inducing muscle force is unknown (Fig 5H).

Based on gene expression changes, then, it appears that JAK inhibition by short-term treatment with tofacitinib *may* impact muscle contractility via salutary effects on calcium-related regulatory processes, re-expression of developmental genes, and post-translational modifications (e.g., ubiquitination) relevant to muscle protein function and degradation (Fig 5I), but the functional roles of these altered genes need to further validated.

### Tofacitinib reduces muscle protein oxidation and ubiquitination and increases smooth muscle MYLK in skeletal muscle and cultured myotubes

**Figure 6.**
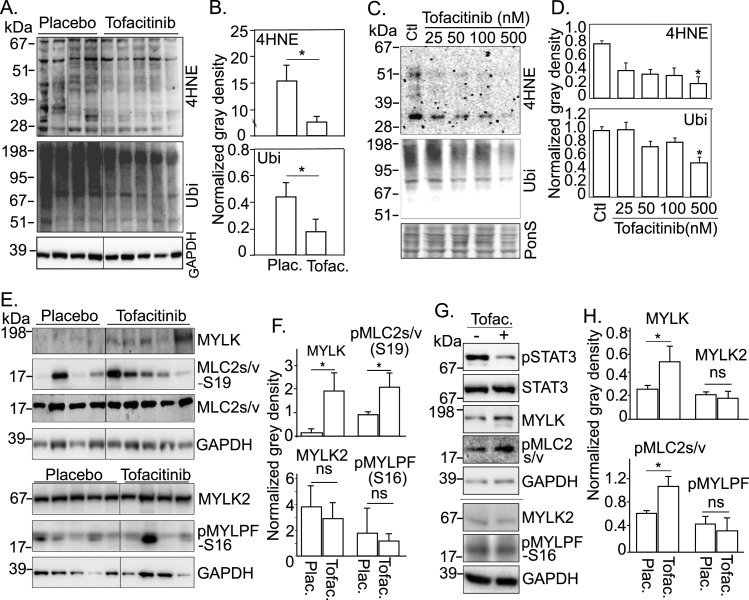
Tofacitinib reduces protein oxidation and ubiquitination and increases the phosphorylation of myosin light chain, in vivo and in vitro. **(A, B)** Tofacitinib suppresses protein oxidation (4HNE) and protein ubiquitination (Ubi) in human diaphragm. **(A)** Western blot, consecutive initial samples at the 10 mg bid tofacitinib dose: placebo n = 4; tofacitinib n = 5. **(B)** Quantitation of gray density of the blotting results, *P < 0.05. **(C, D)** Tofacitinib suppresses protein oxidation (4HNE) and protein ubiquitination (Ubi) in cultured myotubes. Differentiated C2C12 myotubes were treated with tofacitinib doses ranging from 25 to 500 nM for 48 h. **(C)** Western blot shows proteins modified post-translationally by 4HNE and ubiquitination as multiple bands/smears. **(D)** Quantitation of gray density, n = 4/group, *P < 0.05. **(E, F)** Tofacitinib induces the expression of MYLK and the phosphorylation of MLC2s/v but not MYLK2 and phosphorylation of MYLPF, in human diaphragm. **(E)** Western blot, consecutive initial samples at the 10 mg bid tofacitinib dose: placebo: n = 4–5; tofacitinib: n = 4–5. **(F)** Quantitation of gray density, *P < 0.05. **(G, H)** Tofacitinib at 500 nM induces the expression of MYLK and the phosphorylation of MLC2s/v, but not MYLK2 and phosphorylation of MYLPF, in cultured C2C12 myotubes. **(G)** Western blot. **(H)** Quantitation of gray density, n = 4/group, *P < 0.05. Source data are available for this figure.

**Figure S3.**
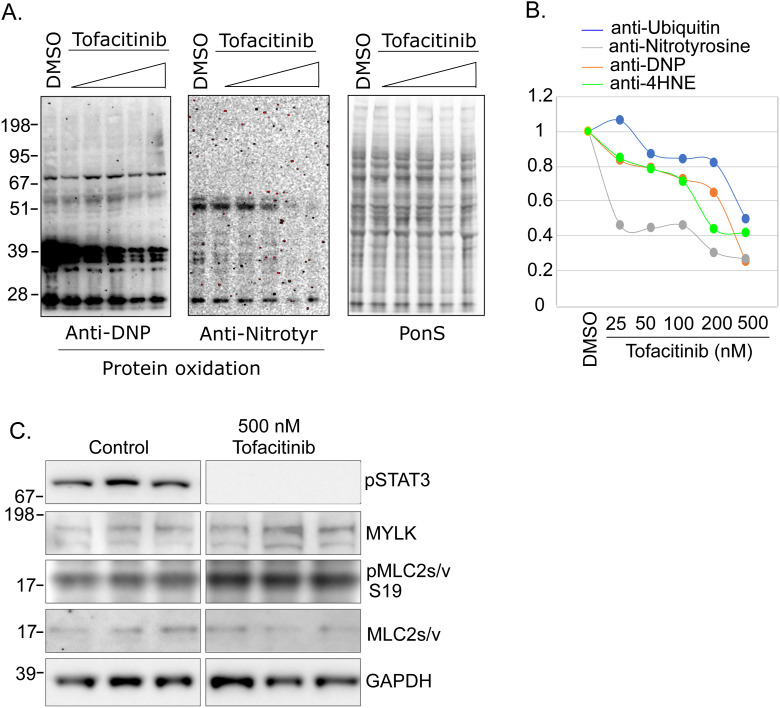
Tofacitinib suppresses protein oxidation and induces MYLK expression and MYL2 phosphorylation in cultured myotubes. Differentiated C2C12 myotubes were treated with 500 nM tofacitinib for 48 h. The protein lysates were extracted from the cultured myotubes and subjected to Western blot analysis (n = 4). ImageJ was used to quantify the gray density from the Western blots. **(A)** Dose-dependent regulation of protein oxidation markers by tofacitinib, ranging from 25 to 500 nM. **(B)** Quantitation of the Western blot by ImageJ. **(C)** Induced expression of MYLK and the phosphorylation of MYL2 (pMYL2-S19 or pMRLC-S19) by tofacitinib. The expression of myosin regulatory light chain (MRLC, or MYL2) was not affected by tofacitinib.

To further understand the regulatory mechanisms responsible for the increased contractile force of tofacitinib-treated muscle, we examined the effect of tofacitinib on muscle proteins. In muscle biopsy lysates, protein oxidation (anti-4HNE) and ubiquitination (anti-ubiquitin) were significantly suppressed in the tofacitinib-treated cohort versus placebo (Fig 6A and B), consistent with the transcriptional down-regulation seen in the ubiquitin ligase conjugation pathway and oxidoreduction systems. To determine if these post-translational modifications of muscle proteins occur as a *direct* effect of tofacitinib on muscle, we examined the effect of tofacitinib in cultured, differentiated myotubes. As shown in Fig 6C and D, increasing concentrations of tofacitinib progressively reduced levels of ubiquitin and 4HNE. Reduction in other protein oxidation markers, such as nitrotyrosine and carbonylation (DNP), was also observed in myotubes following tofacitinib treatment (Fig S3A and B).

Increased MYLK—typically a regulator of *smooth* muscle contraction—was also confirmed at the protein level (∼130 kD) in tofacitinib-treated diaphragm (Fig 6E and F). Concomitantly, the phosphorylation level of slow/ventricular myosin light chain 2/muscle regulatory light chain (MYL2 or MLC2s/v—hereafter called MLC2s/v), was also elevated (Fig 6E and F). In contrast, the typical skeletal muscle type of MYLK, MYLK2, and the phosphorylation level of its substrate MYLPF (myosin light chain 2, fast muscle-type) did not change in response to tofacitinib treatment in the diaphragm (Fig 5E and F). A similar regulatory pattern was seen in tofacitinib-treated, cultured myotubes (Figs 6G and H and S3C). Therefore, inhibition of JAK-STAT activity appears to selectively up-regulate the protein expression of MYLK, and not MYLK2, in skeletal muscle.

### Tofacitinib differentially regulates protein ubiquitination and oxidation and the expression of MYLK between fast- and slow-twitch muscle fibers

**Figure 7.**
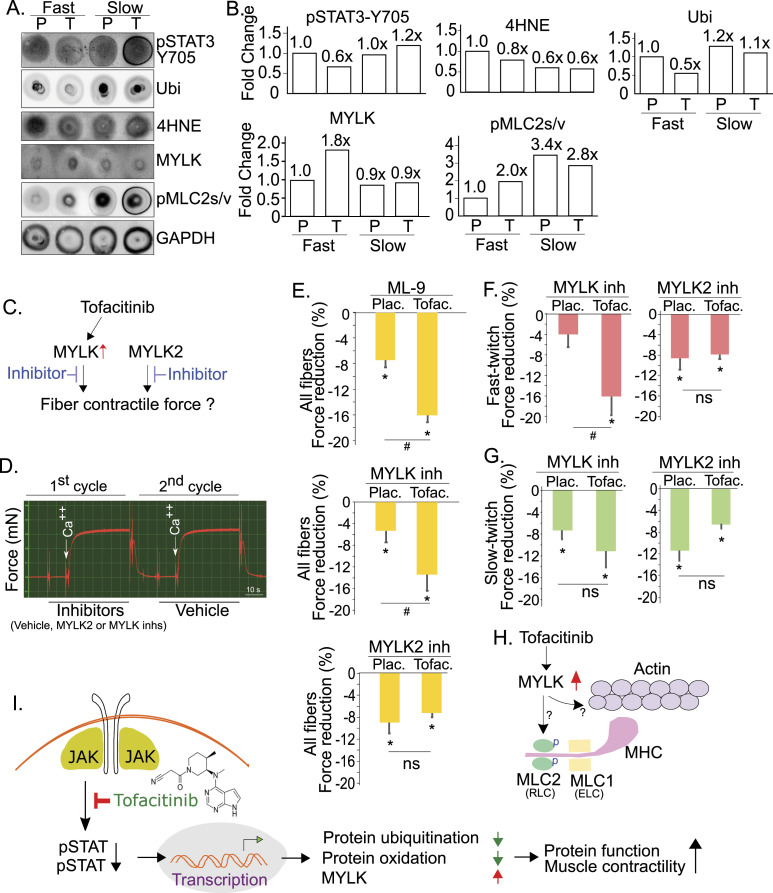
Tofacitinib–up-regulated MYLK is required for force induction in fast-twitch fibers. **(A)** Fiber-specific regulation by tofacitinib examined by dot blot analysis. Protein extracts from four fibers/subject and five subjects/group were pooled for dot blot analyses (P–extracts from placebo-treated fibers; T–extracts from tofacitinib-treated fibers). **(A, B)** Quantitative data with average fold-changes from (A). Note up-regulation exerted by tofacitinib in fast fibers but not in slow fibers. **(C, D)** Schematic diagram to show the strategy and protocol to dissect MYLK’s role in fiber force generation. **(E)** Suppression of MYLK, but not MYLK2, differentially reduced fiber force in placebo- and tofacitinib-treated groups. Inhibitors: ML-9 (1 µm), MYLK inh (peptide 18, 1.5 µm), MYLK2 (1.5 µm) (n = 10–20 fibers. *P < 0.05 for force reduction from baseline (no inhibitor) to the addition of inhibitors; #*P* < 0.05 for differences in the degree of the force reduction between the placebo- and tofacitinib-treated group; ns, no significant difference). **(E, F)** MYLK inhibitor, but not MYLK2 inhibitor, suppressed fiber force more significantly in the tofacitinib group in fast-twitch fibers (n = 5–10 fibers, * and # as in (E)). **(G)** MYLK inhibitor and MYLK2 inhibitor suppressed fiber force in slow-twitch fibers, with no difference between placebo and tofacitinib groups. n = 5–10 fibers, * and# as in (E, F). **(H)** Unknown signaling downstream of MYLK in skeletal muscle. Hypothetically, MYLK may regulate skeletal muscle fiber force through phosphorylation of myosin light chain 2/MLC2 and/or other unknown substrates. **(I)** Summary diagram of the hypothetical mechanisms underlying tofacitinib-dependent increase in contractile force of diaphragm muscle. Both the suppression of protein ubiquitination and oxidation, and the induction of MYLK, likely contribute.

Given our finding that fast-twitch fiber force was increased by tofacitinib but slow-twitch fiber force was not, we examined if the regulatory mechanisms of tofacitinib differs between fast-twitch and slow-twitch fibers. Because of the low protein abundance in individual, short (∼3 mm), myofibers, we pooled 20 fibers from 5 subjects (4 fibers per subject) in each cohort to examine protein expression with dot blotting (Fig 7A and B). As STAT3 activity—that is, pSTAT3 level—was suppressed in fast-twitch fibers by tofacitinib, protein oxidation and ubiquitination markers were reduced, whereas the phosphorylation of MLC2s/v, that is, pMLC2s/v, was increased. Although not abundantly expressed, MYLK was detected in both fast- and the slow-twitch muscle fibers, and it was up-regulated by tofacitinib in fast-twitch fibers. Conversely, STAT3 activity was not suppressed in slow-twitch muscle fibers, MYLK and phosphorylated MLC2s/v, and the levels of the protein ubiquitination and oxidation markers were also not altered in slow-twitch fibers (Fig 7A and B). This result from single fibers further validates the observation of tofacitinib-dependent regulation at the protein level and suggests a possible role of these molecular changes in our finding that force was increased by tofacitinib in fast-twitch but not in slow-twitch fibers (Fig 3). It remains possible that a higher concentration of tofacitinib is required to suppress JAK activity in slow-twitch fibers. The reasons why this might be the case are currently unknown.

### MYLK plays a role in tofacitinib-dependent induction of fiber force in fast-twitch muscle fibers

Because tofacitinib up-regulates smooth muscle-type MYLK and not skeletal muscle-type MYLK2, we hypothesized that MYLK may contribute to the tofacitinib-dependent induction of diaphragm fiber force. To test this idea, we used inhibitors of MYLK and MYLK2 to block each of their activities and examined the resulting force changes in single fibers (Fig 7C). Single muscle fibers were isolated from the tofacitinib-treated and placebo groups, with 10–20 fibers per group. After sequential incubation in pre-activation and activation solutions with ML-9 (competitive small-molecule MYLK inhibitor), MYLK inhibitory peptide (MYLK peptide 18), or MYLK2 inhibitory peptide, respectively, single-fiber force was measured (first cycle shown in Fig 6D). Control (without inhibitor) fiber force was measured after washing off the inhibitors (second cycle shown in Fig 7D). Percentage change of the peak fiber force, with inhibitor versus without inhibitor, was used as an index to compare the effect of each individual inhibitor on fiber force.

Shown in Fig 7E, in all fibers examined, MYLK inhibitor ML-9 was able to reduce the fiber force in both the placebo- and tofacitinib-treated groups, but with a significantly higher level of suppression in tofacitinib-treated fibers. To eliminate the concern that ML-9 may achieve the observed effect through the suppression of enzymatic activities other than MYLK’s, we used another more selective MYLK inhibitor, MYLK peptide 18, and observed a very similar result (Fig 7E). On the other hand, there was no difference in the degree of fiber force reduction between the placebo- and the tofacitinib-treated groups with the MYLK2 inhibitor, although it indeed attenuated the force at the baseline level in both groups (Fig 7E). Furthermore, looking at fast-twitch fibers only (Fig 7F), the MYLK inhibitor, but not the MYLK2 inhibitor, showed more significant suppression of fiber force in tofacitinib-treated fibers. There was no significant difference between the placebo and tofacitinib groups in the slow-twitch–only fibers in the presence of either the MYLK inhibitor or the MYLK2 inhibitor (Fig 7G). Taken together, both MYLK and MYLK2 contribute to skeletal fiber force at the baseline level; however, only MYLK contributes to the increase in force generation induced by tofacitinib in fast-twitch fibers.

## Discussion

Skeletal muscle weakness is a major clinical problem in patients suffering from both primary and secondary muscle disorders. Ventilator-induced diaphragm dysfunction is an example of pathological skeletal muscle weakness, for which successful treatment/prevention would perhaps provide the most immediate benefit—improvement in ventilator weaning, reduced ventilator time, and thus reduced ICU morbidity/mortality. We set out, based on preclinical work by our group and others (Smith et al, 2014; Tang et al, 2015), to investigate whether JAK inhibition improves skeletal muscle force generation in humans. We examined muscle samples from patients taking a short course of the JAK inhibitor, tofacitinib, and compared them with samples from randomized patients receiving a placebo. We found that JAK inhibition substantially increased skeletal muscle fiber contractile force in both diaphragm and serratus anterior muscles—and presumably in skeletal muscle broadly.

To summarize our findings, the administration of five, 10 mg doses of oral tofacitinib over 48 h substantially increased maximal muscle fiber contractile force (by ∼15%) within both the human diaphragm (a respiratory muscle) and serratus anterior (a non-respiratory muscle). This degree of increase in muscle force is not only statistically significant but also highly clinically significant. There is no drug currently regularly used in patients, or considered safe to use, to increase muscle contractility. Theophylline increases diaphragm strength acutely, but it has a narrow therapeutic window and a checkered safety/toxicity record (Murciano et al, 1984; Minton & Henry, 1996) and therefore has not been generally used for this purpose. Anabolic steroids increase muscle strength over only a longer time frame, and they also have serious associated toxicities that dramatically limit their use. Caffeine may improve the strength of some muscles (generally by under 10%) (Grgic et al, 2018), but this remains controversial (Polito et al, 2016). Tofacitinib would thus appear to be the only currently marketed drug that increases skeletal muscle contractile force. Our findings suggest that JAK inhibition may be a useful therapy for at least some of the many clinical situations in which skeletal muscle weakness increases morbidity and mortality—including cancer cachexia, primary neuromuscular diseases, renal failure, and the sarcopenia of aging. One can even envision potential applications outside of healthcare.

Protein ubiquitination and oxidation are known to impact muscle force production by modulating the turnover and function of muscle proteins, and the structure of the contractile apparatus (e.g., the cross-bridge) (Bayeva & Ardehali, 2010; Kumar et al, 2022). Oxidative damage to myofibrillar proteins is thought to impair contractility through disruption of actin-myosin interactions and cross-bridge cycling. Tofacitinib treatment appears to ameliorate these post-translational modifications, consistent with our prior observations of the effect of JAK inhibition on muscle in preclinical models and cultured muscle cells (Tang et al, 2015).

However, the current findings also suggest a second mechanism by which JAK inhibition may increase contractile force—via induction of a smooth muscle type of MYLK. It is known that phosphorylation of myosin regulatory light chain, a process that can be catalyzed by calcium/calmodulin-activated MYLK, is positively associated with muscle force through modulating the mobility of myosin heads and the formation of cross-bridges (Szczesna et al, 2002; MacIntosh, 2003; Stull et al, 2011; Vandenboom, 2016). Typically, MYLK2 is the major isoform of MYLK in skeletal muscle, whereas MYLK is considered to be the predominant smooth muscle isoform of MYLK. However, it has been demonstrated by previous authors that smooth muscle type MYLK is indeed expressed not only in smooth muscle but also in skeletal muscle (Herring et al, 2000) (see Fig 1B in the reference). We show here that MYLK2 is expressed abundantly in skeletal muscle, but MYLK2 and the phosphorylation level of its known substrate, MYLPF (fast muscle type myosin regulatory light chain), were not regulated by tofacitinib in the diaphragm muscle nor in differentiated myotubes in culture (Fig 6E–H). Conversely, the expression of MYLK (∼130 kD) was induced in diaphragm muscle extract. Furthermore, MYLK expression was confirmed in these skeletal muscle fibers (fast and slow) in our isolated single muscle fibers, which *cannot* have contamination by smooth muscle, and it was up-regulated in tofacitinib-treated fast fibers (Fig 7A). Blocking MYLK activity (but not MYLK2 activity) with selective inhibitors attenuated fiber force by a significantly greater degree in tofacitinib- versus placebo-treated fibers, and it had this effect in fast-twitch fibers but not slow-twitch fibers. This provides direct evidence establishing that up-regulation of MYLK contributes to tofacitinib-dependent force induction.

The downstream mechanisms underlying this effect of MYLK remain to be elucidated. Hypothetically, they may include the phosphorylation of the well-known targets: myosin light chain 2, for example, MLC2s/v, because it is indeed present in fast type II fibers (Stuart et al, 2016), and perhaps also other substrates such as troponin isoforms (Sevrieva et al, 2020) (Fig 7H), which will need to be further validated. The current study is focused on the identification of MYLK’s role. It should also be mentioned that although regulation of satellite cell activity is a known and important effect of JAK signaling (Chazaud & Mouchiroud, 2014; Doles & Olwin, 2014; Price et al, 2014; Tierney et al, 2014; Sorensen et al, 2018; Moresi et al, 2019), and myogenesis-relevant Wnt/NOG/bone morphogenetic proteins signalings are activated, the activation of satellite cells would be unlikely to be a relevant mechanism increasing muscle force over the short 48 h time-course during which we administered tofacitinib. An in-depth investigation of the functional roles of these genes in regulating force production in skeletal muscle is necessary in the future.

The interesting observation that tofacitinib differentially regulates fiber force between the fast- and slow-twitch fibers, and the linkage of this to the distinct regulation of protein translational modification (ubiquitination, oxidation, phosphorylation) by tofacitinib in fast- and slow-twitch fibers, indicates that slow-twitch fibers are likely less sensitive to tofacitinib than fast-twitch fibers. The mechanism underlying this sensitivity difference remains to be elucidated, but it is possible that the functioning isoforms of the JAK and STAT families in slow-twitch fibers differ from those in fast-twitch fibers. Perhaps a higher dose of tofacitinib, or other JAK inhibitors, would inhibit JAK-STAT activity in slow-twitch fibers as well.

We identified no increased morbidity or mortality after short-course tofacitinib treatment in these patients undergoing a major surgical procedure during which the muscle biopsies were taken. Whereas long-term treatment with tofacitinib has been shown in a randomized post-market study to modestly increase cancer and perhaps cardiovascular risk (Ytterberg et al, 2022), clinical experience with the drug suggests that short- to medium-term use for debilitating muscle weakness would carry very low risk.

Further study will be required to determine definitively if tofacitinib’s impact on muscle strength may be specific to the particular patient population enrolled in this study. Many subjects providing biopsies, for example, had undergone chemotherapy (although in all cases it had been completed by at least 4 wk before the biopsy). Several chemotherapeutic agents can induce oxidative stress (Azmanova & Pitto-Barry, 2022). If oxidative stress was indeed elevated in these patients, then theoretically the reduction in oxidative post-translational modifications by tofacitinib might be more impactful in them than in non-chemotherapy patients. Unfortunately, only 10 subjects had not received chemotherapy, so we did not have sufficient power to reliably carry out this analysis. Furthermore, patients with cancer cachexia, even in the absence of chemotherapy, may demonstrate elevated cytokine levels. This has generally been seen in metastatic cancer not in the locoregional disease extant in these trial subjects with resectable cancer. In addition, we excluded subjects who had lost >5% of body weight—below the threshold of accepted definitions of cancer cachexia (Bozzetti & Mariani, 2009). Again, however, we had only five subjects in the study who did not have cancer, so again we did not have sufficient power to reliably carry out this analysis. On the issue of diabetes as a potential confounder, our finding that only the non-diabetic group demonstrated significantly greater contractile force with tofacitinib sheds serious doubt on the role of this factor in our findings. It is worth noting that even if tofacitinib’s effect does ultimately prove to be limited to, for example, cancer or chemotherapy-receiving patients, this would still represent a substantial subgroup of those with potentially treatable pathological skeletal muscle weakness.

Lastly, because the outcome of difference in baseline muscle contractile force (pre-MV; first biopsy) between the experimental and placebo groups was not prespecified in the original design of our randomized trial (which was designed for the outcome of reducing VIDD [the *change* in force generation between the early and last muscle biopsies]) using a larger n that has not yet been reached, we felt it important to reassure ourselves that we have a low risk of a false-positive result. The results of our bootstrapping analyses, with both the all-fiber and the fast-fiber groups providing a power to reject the null hypothesis of over 80%, provide a high level of reassurance in this regard.

In summary, skeletal muscle fibers from patients treated with the JAK inhibitor, tofacitinib, for 48 h demonstrated substantially greater maximal contractile force compared with a randomized group of placebo-treated controls. Likely mechanisms underlying this improvement in muscle performance after JAK inhibition include reduction in post-translational ubiquitination and oxidative modification and increased expression of proteins, for example, MYLK, that positively regulate contractility (Fig 7I). Further study with whole muscle force testing and other clinical outcome measures is required before this might be adopted as a widely used therapy. However, the results reported herein offer the strong possibility that JAK inhibition could become the first widely applicable therapy for some of the many disorders characterized by skeletal muscle weakness.

## Materials and Methods

### Human subjects

Human muscle biopsies were acquired from patients participating in an ongoing prospective, randomized clinical trial, which was approved by the Stanford Institutional Review Board and funded by the NHLBI (full study design available in our previous publication) (Shrager et al, 2021) (clinicaltrials.gov #NCT03681275). The goal of the clinical trial as designed is to test the efficacy of tofacitinib in preventing ventilator-induced diaphragm dysfunction (VIDD: i.e., the change in fiber force generation between early and late diaphragm biopsies during the 7-h-long esophagectomy procedure in tofacitinib-receiving versus placebo groups). For the study reported herein, the available muscles from the early biopsies (before any substantial time on mechanical ventilation [MV]) were independently examined to investigate the impact of JAK inhibitor on *baseline* force generation of human skeletal muscle (*not* VIDD). Informed consent was obtained from all subjects and the study conformed to the principles set out in the WMA Declaration of Helsinki and the Department of Health and Human Services Belmont Report.

Subjects were screened based on the following inclusion and exclusion criteria. (A) Inclusion criteria: age 15 or older undergoing esophagectomy. (B) Exclusion criteria based upon potential tofacitinib toxicities/drug interactions: (1) ALT, AST, total bilirubin, or alkaline phosphatase level above 150% of normal (reducing tofacitinib excretion); (2) creatinine level above 2.0 (reducing tofacitinib excretion); (3) currently taking systemic antifungal medication (tofacitinib levels are increased by some antifungals); (4) currently taking systemic immunosuppressive medications (to avoid marked immunosuppression; including corticosteroids, which may impact muscle structure/function and thus confound the study); (5) history of untreated TB, or a positive PPD or positive QuantiFERON test which has not been treated (because tofacitinib can reactivate TB). Exclusion criteria based upon potential but unlikely impact of small diaphragm biopsies on respiratory muscle function (and to reduce confounding conditions): (1) FEV1 or FVC under 70% predicted, or DLCO under 50% predicted (if measured); (2) neuromuscular disease that might compromise diaphragm function. Exclusion criteria based upon general considerations (and to reduce confounding conditions): (1) pregnancy; (2) >5% weight loss over the last 6 mo (which may indicate cachexia, which can be associated with JAK/STAT pathway activation and impact muscle structure/function).

Participants were randomized initially to receive tofacitinib versus identical-appearing placebo, 5 mg, orally bid, a total of five doses, starting 48 h before the surgery (Fig 1A). As per the study plan, after 10 subjects were enrolled, we measured the inhibition of STAT3 phosphorylation in biopsies and discovered that the target pathway was not inhibited. A study amendment was then approved to increase to 10 mg per dose. We report data herein from only those who received the 10 mg dosing.

Diaphragm and serratus anterior muscle biopsies were taken as soon as each of the muscles became surgically accessible at the start of the operation. After harvesting the ∼8 × 8 × 6 mm muscle biopsies, they were sharply dissected into two parts in line with the myofibers. One part was immediately immersed in 4 ml of rigor buffer to permeabilize the myofibers at 4°C, overnight, for later single-fiber force measurement. This part was then transferred to a new vial with 1 ml fresh rigor buffer and stored at −20°C until the time of dissection for single-fiber force measurement, within 300 d of harvesting. The remaining muscle was snap-frozen in liquid nitrogen for molecular and biochemical analyses. All study personnel collecting data and performing both wet laboratory and statistical analyses were blinded to the study group, and patients were blinded to whether they had received tofacitinib or placebo.

### Single-fiber force measurement

Single-fiber force was measured using a Permeabilized Fiber System (1410A; Aurora Scientific). Briefly, individual myofibers were isolated along their longitudinal axis from skinned bundles, minimizing mechanical trauma. Single myofibers were mounted between a force transducer (403B; Aurora Scientific) and a length controller (802D; Aurora Scientific). The myofibers were then adjusted to 2.6 ± 0.1 μm sarcomere length and the diameter and length were measured. The contractile force was measured using a programmed protocol in which the mounted fiber was sequentially immersed in relaxing solution (10 s), pre-activating solution (10 s), and then activating solution (35 s) containing various Ca^2+^ concentrations (range 1–5.5 mM, correlated to pCa 6.6 to pCa 3.3, Table S3) at 15°C, adapted from a protocol previously established by one of the co-authors (Cooke et al, 1988). The fibers rested for 60 s between measurements. Mounted fibers showing any visual signs of damage, contortion, or overstretching are discarded. Fibers with maximal specific force of less than 20 KN/m^2^ are also considered to be damaged and are discarded from the data set. The specific force (KN/m^2^) was calculated by maximum force/[πd^2^/4], in which d = diameter of the myofiber.

Table S3. Conversion between pCa and free calcium for the single fiber force experiments.

To examine the impact of MYLK inhibitors and MYLK2 inhibitors on fiber force generation, the protocol of force measurement was modified. Briefly, after fibers were mounted, the fiber force was first measured (at pCa 5.1) without inhibitors over two consecutive cycles (i.e., first and second cycle, Fig 7D) to determine the baseline change of the peak fiber force between the two cycles. Without inhibitors, the difference in the peak fiber force between the two consecutive cycles was negligible. Next, the individual inhibitor of interest was placed into both the pre-activation solution (10 s) and the activation solution (35 s) for the first cycle of the force measurement; then, after washing off the inhibitors (20 s), we measured the peak fiber force over the second cycle (35 s) in the absence of inhibitors, as the control. The inhibitors used were ML-9 (a small-molecule MYLK inhibitor; MedChemExpress), the MYLK inhibitory peptide (MYLK peptide 18; MedChemExpress), and the MYLK2 inhibitory peptide (Santa Cruz Biotechnologies). The percentage change of the peak fiber force from that measured with inhibitors versus that measured without inhibitors was used as an index to evaluate the effect of each individual inhibitor on fiber force. Fiber force in the presence and absence of these inhibitors was measured from the same fiber to eliminate the impact of force variation from fiber to fiber.

### RNA sequencing

Briefly, total RNAs were extracted from human diaphragm (placebo: n = 4; tofacitinib: n = 3), purified by oligo-dT magnetic beads, and sequenced on an Illumina platform. The reads mapped to each gene were counted by FeatureCounts v1.5.0-p3, and the FPKM (Fragments Per Kilobase of transcript sequence per Million base pairs sequenced) of each gene was calculated. Differential expression analysis between the placebo- and tofacitinib-treated groups was performed using the DESeq2 R package (1.20.0). The resulting *P*-values were adjusted using the Benjamini and Hochberg’s approach for controlling the false discovery rate. Genes with an adjusted *P*-value ≤ 0.05 found by DESeq2 were considered differentially expressed. Enrichment analysis of differentially expressed genes was analyzed by DAVID bioinformatics and Gene Set Enrichment Analysis for molecular events and signaling pathways.

### Dot blotting (fiber typing) and Western blotting

**Figure S4.**
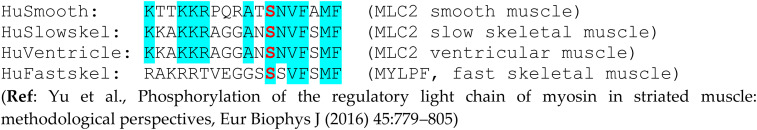
Comparison of the amino acid sequences flanking the phosphorylatable serine (in red) of the myosin light chain 2/regulatory light chain between the isoforms of human smooth muscle (HuSmooth), ventricular muscle (HuVentricle), slow (HuSlowskel), and fast (HuFastskel) skeletal muscle. Note the isoforms from the smooth, ventricle, and slow skeletal muscle but not from the fast skeletal muscle share high similarity.

Fiber type was determined by dot blotting lysates from single fibers after recovery from the force measurement apparatus, with specific antibodies against fast-twitch and slow-twitch myosin heavy chains (Sigma-Aldrich). Fibers were assigned based on their predominate fiber types (slow- and fast-twitch), or as mixed. Western blotting was performed with whole muscle lysates after running on a 4–12% SDS–PAGE gel and transferring to a nitrocellulose membrane. Antibodies were against STAT3, pSTAT3(Y705), 4HNE, pMLC2s/v(S19) (Fig S4), Ubiquitin, and GAPDH (Cell Signaling Technologies); and MLC2s/v, MYLK (Santa Cruz Biotechnologies).

### Statistics

Differences in the mean fiber forces between the two groups at each measured calcium concentration were determined by *t* test. Differences in patient demographics between groups were assessed by the Wilcoxon rank sum test or Fisher’s exact test, as appropriate. Considering that the muscle force does not respond constantly along all calcium concentrations pCa 6.5 to 2.8, piecewise random effect modeling was adopted to estimate the adjusted effects (see details of modeling in supplementary information). For all analyses, a two-sided α-level of 0.05 was considered statistically significant. We planned one prespecified and performed one post hoc, subgroup analysis (see the Discussion section). Analyses were performed using SAS version 9.4 (SAS Institute Inc.) and R Studio: Integrated Development Environment for R (Posit Team 2022).

The entire clinical trial from which this subset of patients/biopsies was drawn, was powered at n = 56 based upon the outcome of prevention of VIDD (i.e., *change* in fiber force generation between the early and late biopsies in tofacitinib-receiving versus placebo groups). No formal, pre-study power analysis was performed for the 33 patients included for the outcome reported in this manuscript—baseline, pre-MV force generation. To establish the low likelihood of type II errors in the results derived from these n = 33 subjects in this report, we performed a post hoc bootstrap analysis. This was performed by resampling our fiber force dataset from the diaphragm and serratus anterior, through the unrestricted random sampling with a replacement scheme, to create the simulated bootstrap samples, which had the same sizes (for both the tofacitinib and the placebo groups) as the original clinical trial dataset. Two-sample *t* test was then used to determine if the mean muscle fiber force of the tofacitinib and placebo groups were significantly different at calcium levels pCa 5.1 on the simulated bootstrap samples. The resampling procedure was repeated 1,000 times, and 1,000 bootstrap samples were generated. For both the all-fiber group, the slow-twitch–only group, and the fast-twitch–only group, we assessed type II error at calcium levels pCa 5.1.

## Supplementary Material

Reviewer comments
