# Supplementary material for: JAK inhibition with tofacitinib rapidly increases contractile force in human skeletal muscle
Source: Life Sci Alliance. 2024 Aug 9;7(11):e202402885. doi: 10.26508/lsa.202402885 (PMC11316201; doi:10.26508/lsa.202402885)
Supplement: Supplementary file 3 [file LSA-2024-02885_SdataF6.pdf]

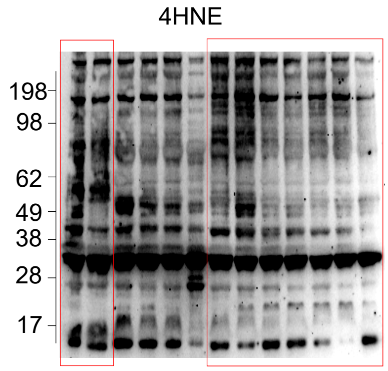

Source file for Fig. 6A

Original western bloting results. Bands in the red boxes are samples from the trial subjects. Samples ran on the same gel from non-trial specimens have been removed.
